# Adrenal cortical carcinoma with extension into the inferior vena cava – case report and literature review

**DOI:** 10.1186/1746-1596-9-51

**Published:** 2014-03-06

**Authors:** Lukasz Fulawka, Dariusz Patrzalek, Agnieszka Halon

**Affiliations:** 1 Department of Pathomorphology and Oncological Cytology, Wroclaw Medical University, ul. Borowska 213, 50-556 Wroclaw, Poland; 2 Department of Pathomorphology, Lower Silesian Oncology Centre, pl. Hirszfelda 12, 53-413 Wroclaw, Poland; 3 Department of Surgery, 4th Military Clinical Hospital in Wroclaw, ul. Weigla 5, 50-981 Wrocław, Poland

**Keywords:** Adrenal cortical carcinoma, Adrenocortical carcinoma, Adrenocortical tumour, Weiss criteria, Van Slooten criteria

## Abstract

Adrenocortical carcinoma (ACC) is a rare endocrine malignancy with an incidence rate of 1 to 2 per million person-years. ACC most commonly arises sporadically, but may be associated with familial tumour syndromes. Clinical symptoms are mainly related to an excess of steroid hormones. We present an unusual case of adrenocortical carcinoma in a 27-year-old male who complained of non specific mass-effect related symptoms of slowly growing intensity differing from others described in literature because of the patient’s age and the sudden deterioration of the clinical course. The tumour was resected with the left kidney with an extension into the inferior vena cava. Histological examination revealed morphological features characteristic of an adrenal cortical tumour. The immunohistochemical results (positive reactions for vimentin, CD56, inhibin, melan A, synaptophysin, bcl-2, calretinin) confirmed the diagnosis. According to the most widely used modified Weiss criteria and the Van Slooten system, a diagnosis of adrenal cortical carcinoma was strongly confirmed. The postoperative condition was poor. Reoperation was conducted, including abdominal aorta thrombectomy and aortic prosthesis implantation. The patient died two days after the second operation. Autopsy revealed a metastatic tumour in the left lung and morphological symptoms of acute circulatory collapse due to a massive haemorrhage into the abdominal cavity, which was the direct cause of death.

**Virtual slides:**

The virtual slide(s) for this article can be found here: http://www.diagnosticpathology.diagnomx.eu/vs/1602226377106882.

## Background

Adrenocortical carcinoma (ACC) is a rare endocrine malignancy with an incidence rate of 1 to 2 cases per million person-years [1-3]. It most commonly occurs in two age groups: the under fives, and 40–59, and is twice as common in women [1-6]. Most ACC cases are sporadic. However, familiar neoplasms also exist, most commonly associated with multiple endocrine neoplasia type 1 (MEN-1), Li-Fraumeni syndrome, Beckwith-Wiedemann syndrome and Carney complex [1,6].

Clinical symptoms are related to hormonal dysfunction, which occurs in 60–80% of cases [1,4,6]. Most commonly it is glucocorticoid or both glucocorticoid and androgen hypersecretion [1,4,6]. Glucocorticoid-secreting adrenocortical tumours are responsible for the minority (10–15%) of endogenous Cushing syndrome [7]. It is manifested by central obesity, skin-thinning, muscle atrophy, osteoporosis, immunodeficiency, diabetes and hypertension [1]. Androgen excess in women results in virilization. Cases secreting only or predominantly androgen are not uncommon [1]. Estrogen oversecretion may lead to gynecomastia in men and metrorrhagia in postmenopausal women. Exceptionally, ACC produces aldosteron leading to hypertension and hypokalaemia [1,4,6]. Cases with nonfunctional tumours may have mass-effect related symptoms or may be discovered as incidentalomas [1,6].

ACCs are generally large tumours, with an average diameter of 5–20 cm and mass of 1000 g [1,2,6]. According to some authors, they are most commonly diagnosed as an advanced disease (stage IV), with tumour mass extending beyond the adrenal gland and metastases (most commonly lungs, liver or lymph nodes) [1,6,8]. Five-year survival of patients with ACC varies from 30 to 45% [2].

In this paper we report the case of an adrenocortical carcinoma extending into the inferior vena cava and metastasis to a lung, with a fatal outcome.

### Clinical presentation and management

**Figure 1:**
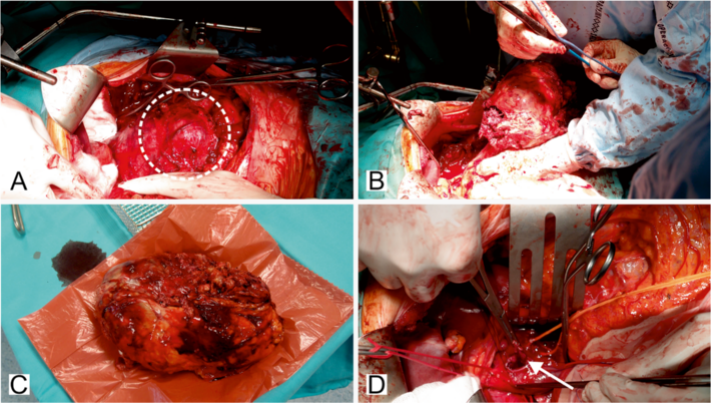
**Surgical operation. (A)** Large mass of the left adrenal gland revealed in laparotomy (circle). **(B)** Tumourectomy. **(C)** Tumour after resection. **(D)** Thrombectomy of the inferior vena cava (arrowed).

A 27-year-old male with insulin-dependent diabetes had been complaining of intensifying stomach fullness, pain and nausea for two years. The patient was admitted to a surgery ward with a radiologically identified tumour of the left kidney and adrenal gland.Laparotomy was conducted by excision under the left costal arch. A large adrenal gland tumour was revealed (Figure 1A). The left kidney and renal vessels were melted into the tumour mass, and the spleen was bound to it. The whole tumour was resected with the left kidney (Figures 1B, 1C). A splenectomy was performed, and a thrombectomy of the inferior vena cava was conducted (Figure 1D).

After surgery the patient was transferred to an intensive care ward with symptoms of MODS (multiple organ dysfunction syndrome). Despite intensive medication (catecholamines infusion, mechanical ventilation, diuretics infusion, fluid replacement, blood transfusion and targeted antibiotic therapy), no improvement in clinical condition was observed. A computed tomography angiography revealed critical obstruction of the abdominal aorta. An urgent relaparotomy was conducted. This was followed by abdominal aorta thrombectomy and aortic prosthesis implantation. After surgery, the patient’s clinical condition continued to deteriorate, and he died two days after the second operation.

### Macroscopy of surgical specimen

A surgical specimen was taken which measured 22 × 17 × 10 cm (Figure 1C). The fat tissue adjacent to the superior pole of the left kidney revealed a solid grey-yellow tumour measuring 17 × 12 cm, which disintegrated on palpation. Serial sectioning of the borderline between tumour and kidney showed the tumour mass reaching the fibrous capsule of the kidney without evident infiltration of the kidney cortex. Sections of the vessels contained emboli. Besides the tumour mass, other surgical specimens were examined. The first contained the tumour thrombus from the inferior vena cava. The second contained paraaortic and retroperitoneal lymph nodes. There were 8 lymph nodes in total.

### Autopsy results

On autopsy, 200 ml of haemorrhagic fluid was revealed in the peritoneal cavity. The peritoneal surface was covered with numerous thrombi. Condensation of thrombi was apparent in the area of tumour bed and aortic prosthesis. Dissection of the inferior vena cava revealed a grey mass bound to its wall in the area of prior surgical excision. The left pleural cavity contained 1000 ml of yellowish transparent fluid, the right pleural cavity 600 ml. On pressure, pinkish-yellow slightly foamy liquid came out from sectional area of both lungs. Left lung dissection revealed a solid whitish well-circumscribed tumour of 2 cm in diameter in the superior lobe. The bronchi of both lungs contained a thick transparent secretion. Dissection of the pericardial cavity revealed 80 ml of yellowish transparent fluid. The brain surface showed flattened gyri and obliteration of sulci.

To summarize, the autopsy revealed symptoms of acute circulatory collapse due to massive haemorrhage from the tumour bed and from the site of the aortic prosthesis implantation.

### Histopathology

**Figure 2:**
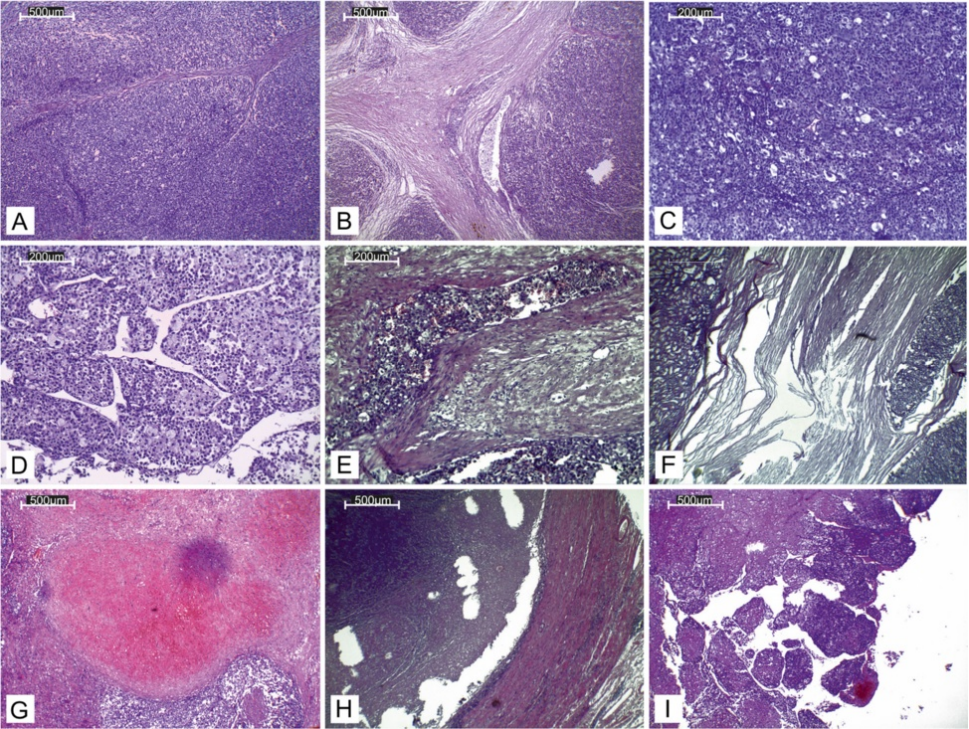
**Histopathology. (A)** Predominant solid area consisted of cells separated by fibrous bands forming fine lobules. **(B)** Broad fibrous bands dividing the tumour into prominent lobules. **(C)** Nest of cells separated by a fine vascular network. **(D)** Clear-cytoplasm cells forming broad trabeculae. **(E)** Capsular invasion. **(F)** Tumour infiltrating renal capsule without renal tissue invasion (renal cortex visible on the left). **(G)** Central part of the tumour with a large haemorrhage. **(H)** Medium-diameter artery cross-section obliterated with necrotic tumour mass. **(I)** Thrombus from the inferior vena cava consisting of tumour mass with necrosis, intermingled with fibrin.

**Figure 3:**
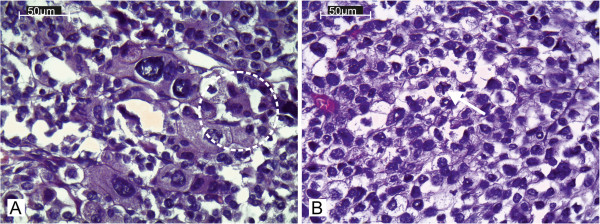
**Histopathology-cytologic features. (A)** Marked nuclear atypia manifested by significant anisokaryosis and poikilokaryosis. Monster cells with large nuclei. A few of them create syncytial structure. **(B)** Atypical mitosis (arrowed).

**Figure 4:**
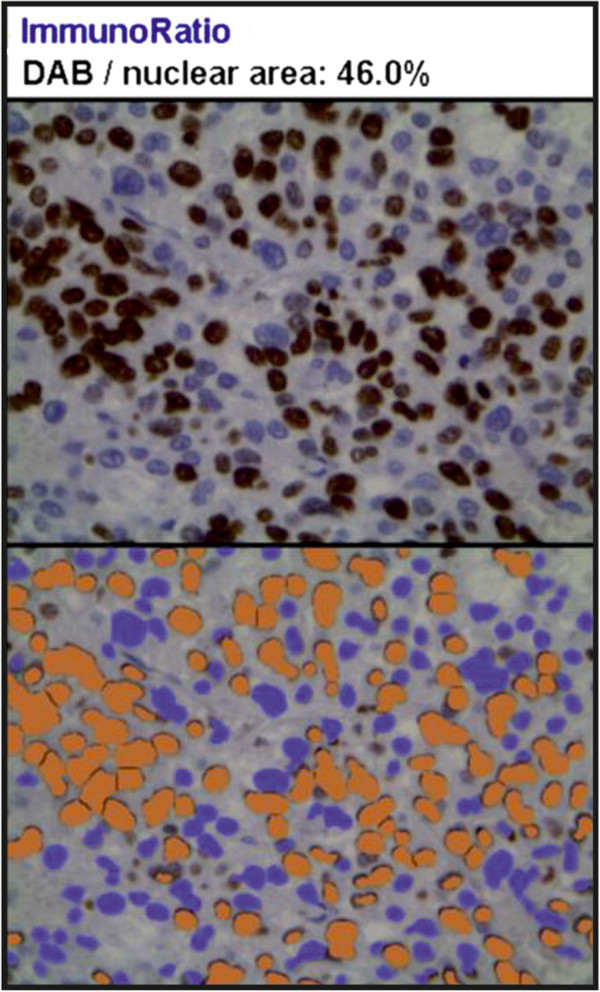
**Ki67 immunohistochemistry.** Proliferation index, PI = 46% (assessed with ImmunoRatio application [9]).

On scanning view the tumour architecture was predominantly solid (Figure 2A). Barely visible fibrous bands separated the tumour mass into fine lobules. Considerable area was divided by broad fibrous bands into prominent lobules (Figure 2B). Medium magnification revealed a nests of cells separated by a fine vascular network (Figure 2C). A minority of the tumour area represented a broad-trabecular pattern (Figure 2D), and was predominantly composed of clear-cytoplasm cells. Overall, clear cells made up no more than 25% of the whole tumour area. Capsular invasion was visible (Figure 2E). The tumour infiltrated the renal capsule without renal tissue invasion (Figure 2F). Necrosis occupied a significant area of the tumour. In the central part of the tumour a large haemorrhage was visible (Figure 2G). Small- and medium-diameter vessel cross-sections unveiled obliteration with necrotic tumour masses (Figure 2H). High-power magnification revealed significant atypia manifested by prominent anisokaryosis and poikilokaryosis (Figure 3). The nuclei were hyperchromatic with coarse chromatin. There were a few foci of monster cells with huge nuclei, a few of them forming syncytia (Figure 3A). The proliferation index (PI) was 46% (assessed with ImmunoRatio web application [9], Figure 4). Mitotic index (MI) was 22 per 10 high power fields (HPF). A few atypical mitoses were encountered (Figure 3B).

**Figure 5:**
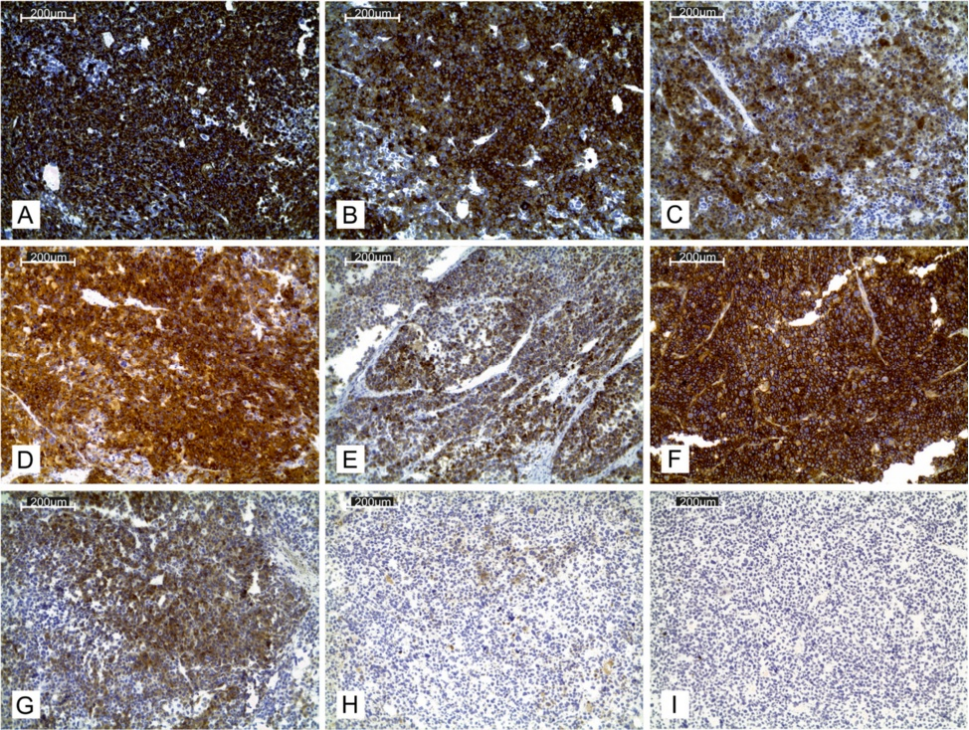
**Immunohistochemistry. (A)** Vimentin. **(B)** Synaptophysin. **(C)** Calretinin. **(D)** Inhibin. **(E)** Melan A. **(F)** CD56. **(G)** Bcl2. **(H)** Chromogranin A. **(I)** S100.

The tumour cells showed strong diffused immunoreactivity for vimentin, synaptophysin, CD56 and inhibin (Figure 5). A significant number of cells were moderate-to-strong calretinin positive and moderately Bcl-2 positive. Melan A expression was diffusely positive. A few scattered cells among the tumour mass revealed weak chromogranin A immunoreactivity (Figure 5H). The tumour cells were EMA and S100 negative.Histopathological examination of the thrombus from the inferior vena cava revealed a tumour mass with necrosis, intermingled with fibrin (Figure 2I). Sections of the regional lymph nodes showed reactive changes without any metastases. Microscopic examination of the lung tumour revealed a solid pattern similar to the predominant area of the primary tumour with cell nests and a vascular network.

## Discussion

In this paper we present the case of an adrenal cortical carcinoma in a 27-year-old male. According to the literature peak incidence occurs in the forties and fifties [6]. Family history of endocrine malignancy was negative, and so genetic tests for hereditary syndromes associated with increased risk of adrenal cortical malignancy were deemed unnecessary. Our case probably belonged to the predominant group of sporadic ACC. The patient presented symptoms relating to mass effect (stomach fullness, pain and nausea). The same manifestations have been reported by other authors [4,5]. There were no signs of adrenal gland hormonal dysfunction as occurs in 60–80% of cases [1,4,6]. The patient had suffered from insulin-dependent diabetes. However we were unable to find any pathogenetic link between diabetes mellitus type 1 and non-functional ACC in the literature.

Our surgical specimen measured 22 cm at the largest diameter, consistent with that described previously [1,2,6]. The tumour thrombus was present in the inferior vena cava. According to Icard et al., vena cava thrombectomy was conducted in 6% of 253 cases [2]. The probable source of the thrombus were invaded suprarenal and renal veins containing tumour mass on pathological examination.

According to AJCC [10] the tumour was classified as pT3. This category encompasses tumours of any size with local invasion, not invading adjacent organs. In this case the tumour infiltrated its own capsule and the renal capsule but without invading the renal cortex. The N category was staged as pN0 with no metastases revealed on microscopic examination of regional lymph nodes. Autopsy revealed a tumour in the superior lobe of the left lung. Microscopically the lesion revealed a solid pattern, consistent with the primary tumour histology. Thus the category pM1 was applied. Summarizing, the case was placed into stage IV, according to AJCC [10]. There are discrepancies in the statistics quoted by different authors. According to Correa et al. cited by the WHO Classification of Tumours [1,8] the majority (40%) of ACC cases are diagnosed as a stage IV disease. Lafemina states that 61% of ACCs are stage IV [6]. On the other hand, 21.3% of 253 cases in the Icard study [2] were stage IV, while 49.8% were stage II.

The most common adrenal gland tumours are metastatic, as it is the fourth most frequent site of malignancy spread [1]. The most common sources are primary tumours of breast, lung, kidney, stomach, pancreas, ovary and colon [1]. It is therefore important to take any secondary malignancy into account during the initial examination of an adrenal tumour. Most are easy to distinguish on routine hematoxylin-eosin staining. However, some metastatic tumours can mimic adrenocortical neoplasm. Renal cell carcinoma is of special interest, because its pattern is often similar to that of an adrenocortical tumour. In this regard a panel of immunohistochemical assays should be implemented in every suspected adrenal tumour case. This will also help distinguish adrenal cortical from medullary tumours.

On scanning magnification the tumour had a lobular structure with large solid areas separated by broad fibrous bands, consistent with that described as characteristic for ACC [1]. The other typical structures found in our case were broad trabecules. Under medium magnification the solid areas turned out to be nests of cells separated by a fine vascular network, a feature characteristic of ACC [1].

To confirm the diagnosis, immunohistochemical assays were conducted (Table 1). Tumour cells were vimentin, synaptophysin, calretinin, inhibin, CD56 and melan A-positive, consistent with the literature [1,11-15]. A significant number of cells revealed moderate bcl-2 immunoreactivity. There are discrepancies in bcl-2 expression quoted in the literature. Fogt et al. saw a bcl-2 reaction in all 14 adrenocortical carcinomas [16]. Meanwhile, a newer study conducted by Zhang et al. produced almost the opposite results, with 3 bcl-2 positive ACC cases out of total of 10 [15]. A few scattered cells among the tumour mass revealed a weak chromogranin-A reaction. The accessible sources all state consistently that chromogranin A immunoreactivity is negative in all cases of adrenocortical tumours [1,11]. The tumour cells were EMA-negative, consistent with the literature [1,11].

**Table 1:** Immunophenotype of our case compared to the literature

**Immunohistochemistry**	**Our case**	**Data in the literature**	**References**
Vimentin	**(+)**	**(+)**	[11]
Synaptophysin	**(+)**	**(+)**	[11]
Calretinin	**(+)**	**(+)**	[1,15]
Inhibin	**(+)**	**(+)**	[1,11,12,15]
Melan A	**(+)**	**(+)**	[1,11,13,15]
CD56	**(+)**	**(+)**	[11]
Bcl-2	**(+)**	**(+)**	[11,16]
		**(+)** in 30% of cases	[11,15]
Chromogranin A	**(−/+) -** focally	**(−)**	[1,11]
EMA	**(−)**	**(−)**	[1,11]

There are still no unequivocal recommendations to distinguish benign and malignant adrenocortical tumours. A few systems encompassing histological criteria exist. The most commonly appreciated are Weiss criteria modified by Aubert [17]. Our case fulfilled all the criteria, which was significantly beyond 3, the threshold for identifying malignant behaviour (Table 2). Another method for distinguishing adrenocortical adenoma and carcinoma is the Van Slooten system [18]. All the criteria were fulfilled (Table 3). Summing all the values, the total score was 28.4, significantly higher than the score of 8 required to diagnose a malignant adrenocortical tumour.

**Table 2:** **Weiss criteria with Aubert’s modifications for distinguishing adrenal cortical carcinoma from adenoma**[17]

**Weiss criteria**	**Our case (“+” = presence; “-” = absence)**
Nuclear grade by Fuhrman (III/IV)	**+**
MI (mitotic index) >5/50 HPF	**+**
Atypical mitoses	**+**
Clear cells <25%	**+**
Diffuse architecture >33%	**+**
Necrosis	**+**
Venous invasion	**+**
Sinusoid invasion	**+**
Capsular invasion	**+**
**Total Weiss score**	**9**

**Table 3:** **Van Slooten criteria for distinguishing a malignant adrenal cortical neoplasm from a benign one**[18]

**Van slooten criteria**	**Value**	**Our case (“+” = presence; “-” = absence)**
Regressive changes (necrosis, haemorrhage, fibrosis, calcification)	**5.7**	**+**
Lack of normal structure	**1.6**	**+**
Moderate to marked nuclear atypia	**2.1**	**+**
Nuclear hyperchromasia	**2.6**	**+**
Abnormal nucleoli	**4.1**	**+**
MI (mitotic index) >2/10 HPF	**9.0**	**+**
Capsular/vascular invasion	**3.3**	**+**
**Total Van Slooten score**		**28.4**

Histologically, our case presented highly atypical features with deeply pleomorphic areas. In this respect, it was important to search scrupulously for possible sarcomatous differentiation. To our knowledge, nine cases of adrenocortical carcinoma containing a component of sarcoma have been published [3]. Spindle-cell differentiation, consistent with sarcomatous differentiation, was not revealed in our case.

## Conclusions

In this paper we report the case of an adrenocortical carcinoma (ACC) in a 27-year-old patient, a rare malignancy usually occurring in the 40–59 age group. Clinically it was characterized by non-specific symptoms of slowly growing intensity related to mass effect. On the other hand, pathological examination revealed a high-stage metastatic tumour of high malignancy. The postoperative condition was poor. A relaparotomy was conducted, including abdominal aorta thrombectomy and aortic prosthesis implantation, but the patient died two days after the second operation. The direct cause of death was acute circulatory collapse due to a massive haemorrhage into the abdominal cavity as revealed by autopsy.

### Consent

Written informed consent was obtained from the patient’s family for publication of this Case Report and any accompanying images. A copy of the written consent is available for review by the Editor-in-Chief of this journal.

## Competing interests

The authors declare that they have no competing interests.

## Authors’ contributions

LF and AH performed the histological examination and wrote most of the article. DP was the surgeon who operated on the patient, interpreted the patient data, described the surgical procedure and took the photographs. All three authors approved the final text.
